# Medical tourism and national health care systems: an institutionalist research agenda

**DOI:** 10.1186/s12992-018-0387-0

**Published:** 2018-07-16

**Authors:** Daniel Béland, Amy Zarzeczny

**Affiliations:** Johnson Shoyama Graduate School of Public Policy, 101 Diefenbaker Place, Saskatoon, SK S7N 5B8 Canada

**Keywords:** Health care, Medical tourism, Institutions, Institutionalism, Canada, United States

## Abstract

Although a growing body of literature has emerged to study medical tourism and address the policy challenges it creates for national health care systems, the comparative scholarship on the topic remains too limited in scope. In this article, we draw on the existing literature to discuss a comparative research agenda on medical tourism that stresses the multifaceted relationship between medical tourism and the institutional characteristics of national health care systems. On the one hand, we claim that such characteristics shape the demand for medical tourism in each country. On the other hand, the institutional characteristics of each national health care system can shape the very nature of the impact of medical tourism on that particular country. Using the examples of Canada and the United States, this article formulates a systematic institutionalist research agenda to explore these two related sides of the medical tourism-health care system nexus with a view to informing future policy work in this field.

## Background

In this era of globalized medicine, when international travel and access to online health information are readily accessible, medical tourism is an important issue both for national health care systems and from a global health perspective [1–3]. Patients from countries around the world are exercising increasing degrees of autonomy over their health care options by obtaining information from sources other than their regular health care providers and, in some cases, by electing to pursue care alternatives outside their domestic medical system. Medical tourism is a broad and inclusive term that captures a wide range of diverse activities [3]. It has been defined as “the practice of travelling to another country with the purpose of obtaining health care (elective surgery, dental treatment, reproductive treatment, organ transplantation, medical checkups, etc.),” and is generally distinguished from both care sought for unplanned medical emergencies that occur abroad and from formal bi-lateral medical trade agreements [4, 5]. Individual motivations for engaging in medical tourism vary widely and may include imperatives such as avoiding wait times, reducing costs, improving quality, and accessing treatments not available or legal in the home jurisdiction, or for which the individual is not eligible [5–8].

While medical tourism is far from new, shifting patient flow patterns and a growing recognition of the complex ethical, social, economic, and political issues it raises are underscoring renewed efforts to understand this phenomenon and its future [3, 9, 10]. Some of the current attention focused on medical tourism concerns its implications and potential risks for individual patients and health care systems [11–13]. Medical tourism impacts both importing and exporting health care systems, albeit in different ways [14]. Various terms exist to describe trade in health services [15]. For the purpose of this discussion, we will use importing or destination to describe systems whereby patients come from other jurisdictions to receive care, and exporting to describe the departure of individuals from their domestic medical system to pursue health services elsewhere. Recognizing that there are important knowledge gaps and a need for definitional clarity and further empirical work to understand the effects of medical tourism on the countries involved [16], concerns for importing or destination systems include, though are not limited to, ethical questions about inequity of access for local residents versus high paying visitors and about the “brain drain” of local talent into private, for-profit organizations focused on non-resident care [15]. Conversely, the issues exporting systems face often revolve around implications for domestic health care providers, the potential for patients to avoid domestic wait lists, and the costs of follow-up care upon patients’ return [12]. For example, research from Alberta, Canada, suggests that the financial costs associated with treating complications from medical tourism for bariatric surgery are substantial, and complication rates are considerably higher than similar surgeries conducted in Alberta (42.2–56.1% versus 12.3% locally) [6].

Although a growing body of literature has emerged to study medical tourism and address the policy challenges it creates for health systems [3, 16], the comparative scholarship on medical tourism remains too limited in scope, a remark that should not hide the existence of a number of recent comparative studies in the field [17–19]. These studies demonstrate that comparative research is helpful in identifying both the unique and the most common policy challenges facing each country [20] and can, if done appropriately, offer learning opportunities [21]. Indeed, this process can facilitate policy learning (related terms include lesson drawing, policy transfer, diffusion, and convergence) whereby ideas, policies, or practices (e.g., regulatory tools) in one jurisdiction inform or shape those in another [22, 23].

With a view to ultimately informing policy related to medical tourism, this article discusses the value of a comparative research agenda about medical tourism that stresses the multifaceted relationship between medical tourism and the institutional characteristics of national health care systems. On the one hand, these characteristics may shape the content of the demand for medical tourism among the citizens of a particular country [24]. From this perspective, as argued, existing typologies of health care systems can shed light on the varying features of the demand for medical tourism across countries. In other words, different types of health care systems are likely to produce different configurations of demand for medical tourism, which influences the range of policy instruments available to governments and other actors seeking to influence decision-making and behavior within their particular context [25]. On the other hand, the institutional characteristics of each national health care system may also shape the very nature of the impact of medical tourism on that system. Accordingly, the institutional characteristics of health care systems, such as insurance structures [26], may impact both citizens’ demand for medical tourism and the ways in which medical tourism affects each country. Obtaining a better understanding of these relationships may inform new ways of thinking about both the challenges and opportunities medical tourism presents. As medical tourism markets continue to grow and diversify, and as domestic health care systems increasingly feel the stress of limited resources, this kind of work will be critical to support policymakers and health system leaders in their efforts to mitigate the potential harms of medical tourism while, at the same time, responding to the needs of the citizens they serve [3].

Using the examples of Canada and the United States (US), this article proposes the use of an institutionalist research agenda to explore these two related sides of the medical tourism-health care system nexus as a central element of future policy strategies. We first take a comparative perspective on medical tourism and present what we see as key aspects of the issue from a policy perspective. Drawing on current evidence and leading literature in the field, we highlight ways in which national health care systems shape the demand for medical tourism and then, in turn, how medical tourism impacts national health care systems. From this discussion, we identify four key lines of enquiry that we suggest are of critical importance in the medical tourism policy landscape and propose an agenda for future comparative research on medical tourism and national health care systems that could play an important role in informing future policy decisions in this area.

## Medical tourism in comparative perspective

Although gathering robust data on the magnitude of medical tourism continues to be a challenge and more empirical work in this area is needed [3, 5, 10, 12], a strong body of literature addresses different aspects of the issue. For example, research is improving understandings of how medical tourism impacts destination and departure jurisdictions [16, 27], affects relationships with domestic health care providers [28], relates to economic factors including health system costs [29], and impacts clinical outcomes for patients [30], among other important lines of enquiry. However, much of this valuable scholarship focuses on particular forms of medical tourism in specific contexts (bariatric surgery [31], dental care [32], reproductive services [33], etc.) or on the policy and health system implications for individual jurisdictions [13]. There is an increasing amount of comparative research exploring how different features of health care systems may in some cases help drive demand for medical tourism and in other cases constrain it (i.e., push/pull factors), and how they relate to the impact of medical tourism [24], but more work remains to be done in this important area [4, 10]. The potential value of data on the impact of medical tourism in one jurisdiction to structurally- similar systems (e.g., other universal public health care systems) has already been recognized [34]; we agree and suggest that going further with an associated analysis considering the role of their institutional features is critical. This approach is particularly valuable from a policy perspective, especially when it comes to maximizing opportunities for policy learning from other jurisdictions and to identifying and evaluating the respective strengths and limitations of different policy options for decision-makers seeking to, for example, discourage particular forms of medical tourism (e.g., organ transplant tourism [35]).

The governance of medical tourism in its various forms is complex and highly fragmented given its broad range of influential stakeholders (both state and non-state, individual and institutional), its international market-based nature, and its engagement of vastly different and often competing priorities and interests (e.g., profit-driven, patient care, autonomy, ethics, etc.). As a result, policy makers and health system leaders face considerable challenges when it comes to seeking to influence medical tourism markets, whether by encouraging their development or restricting access to them. Obtaining a better understanding of the institutional forces that shape the demand for, and impact of, medical tourism—and connecting those forces to the policy context—may help identify a broader range of tools and options decision- makers can employ to achieve their particular objectives with respect to medical tourism.

Looking at Canada and the US is an appropriate starting point for this comparative work and we use this comparison to ground our analysis of the value of an institutional research agenda as a policy strategy for addressing potential concerns and opportunities associated with medical tourism. While these neighboring countries are similar in many ways, there are dramatic differences in important institutional features of their respective health care systems, including funding and delivery models. The US is both an established importer and exporter of medical tourists, the latter supported in part by insurers offering medical tourism coverage in an effort to reduce the high costs associated with domestic health care services [11, 36]. In contrast, the structure of Canada’s largely publicly-funded, single-payer medical system limits foreign access to non-emergent care and makes it challenging for Canadians to be reimbursed for care received abroad via medical tourism [7]. It also makes the current involvement of Canadians in medical tourism [37] a public policy issue because of its implications for the public purse.

## How national health care systems shape demand for medical tourism

Because health care systems can be understood as relatively stable institutional settings that shape human behavior [38, 39], their features are likely to impact the demand for medical tourism in a particular country or even, in the case of decentralized health care systems subject to considerable regional variation, in a particular region. Health care systems can vary greatly from one country to the next, or even from one region to the next within the same country. Accordingly, what citizens might be looking for when they seek medical treatment abroad is likely to fluctuate based on the nature of health care coverage, financing, and regulation they have at home. Research about these and other drivers is growing but important gaps in knowledge remain [5]. In other words, alongside factors like geographical mobility and travel costs, the institutional configurations of health care systems likely shape, at least in part, the types of services people are looking for based on what health services they can access in their home country, with what degree of quality and timeliness, and at what cost [24].

A comparison between Canada and the US is illustrative here. Starting with the Canadian context, universal coverage has existed in Canada since the early 1970s [40, 41]. Under this framework, regardless of the province or territory in which they live, Canadian citizens and permanent residents are entitled to medically necessary health care services with no user fees, which are strictly prohibited under the 1984 Canada Health Act (CHA). Yet, although the CHA mandates comprehensive coverage for “all insured health services provided by hospitals, medical practitioners or dentists,” many services do not fall under this umbrella and the Canadian health care system has long waiting lists for many non-emergency surgeries like hip replacement [40, 42]. Wait times vary from province to province but they are a source of frustration for many Canadians, some of whom elect to go abroad to get their non-emergency procedure done faster, even if they have to pay for it themselves, instead of relying on the slower public system back home [7]. Gaps in coverage within the single-payer system in important areas such as prescription drugs [43] and dentistry [44] also sometimes push Canadian citizens and permanent residents to go elsewhere for care to reduce costs. There are also a wide variety of medical treatments and health-related interventions offered in private markets that are either not available or not publicly funded in Canada. There are a variety of reasons for this lack of public funding, including those related to evidence (or, more precisely, the lack thereof) regarding safety and efficacy. For example, there is a large international market for unproven stem cell interventions that are not part of the approved standard of care in Canada or available in the publicly funded health care system [45]. Therefore, key motivations underlying the pursuit of Canadian medical tourism often relate to a desire to access care faster, to reduce out of pocket costs for care not covered by provincial health insurance, and/or to access options that are not available in Canada [7].

In the US healthcare system, where about 9% of the population remains uninsured despite the enactment of the Affordable Care Act (ACA) in 2010 [46], people who lack insurance coverage but who face a medical need might go abroad to seek cheaper treatment. In fact, the high cost of care in the US has been recognized as a major factor pushing Americans to seek care at lower cost outside the US, an option that is facilitated by health care globalization [2]. For example, there is research documenting the strong market in the Mexican border city of Los Algodones for Americans seeking dentistry, optometrist, and pharmacy services [47]. Others may be motivated to return to systems with which they are more familiar, as is the case with the Mexican diaspora [24]. In the US, in contrast to Canada where universal coverage prevails, the lack of health care coverage is likely to be a key factor driving the demand for medical tourism. At the same time, waiting times are much less likely to drive the demand for medical tourism in the US, where waiting lists are less of an issue [40].

These brief remarks highlight how key institutional features in both Canada and the US shape patterns in the demand for medical tourism in these two countries, creating both similarities and differences between them. At the same time, regional differences in health system institutions within the two countries can also shape the demand for medical tourism within their borders. For instance, in states like Texas, where elected officials have thus far refused to expand Medicaid as part of the ACA [48], more people live without health care coverage than elsewhere (about 18% of the population as of March 2016 [49]), which may push them to look to Mexico for cheaper health care. Here the institutional characteristics of a state’s health care system and the geographical proximity to Mexico, coupled with the presence of a large population of Mexican descent who speak Spanish, are likely to favor cost-saving medical tourism from Texas to Mexico. This example highlights how geographical and even ethno-cultural factors can shape medical tourism alongside and even in combination with the institutional features of a particular health care system. This is also the case when we deal with issues such as dental care and cosmetic surgeries, which are not covered by many US public and private insurance plans [50].

## How medical tourism impacts national health care systems

At the most general level, existing national and sub-national institutions may mediate the impact on particular countries of transnational processes stemming from globalization [20, 51]. This general remark also applies to global medical tourism, which is unlikely to affect all national health care systems in the same way. Put bluntly, systems will react differently to external pressures, based in part on their own institutional characteristics. Those same institutional characteristics also form part of the policy matrix that shapes the options available to decision makers.

There are two central aspects to this story. First, we can look at how domestic health care institutions are specifically impacted by inbound medical tourism (i.e., destination countries at the receiving end of medical tourism). Research suggests that the way in which health care systems cope with foreign users, and what impact those foreign users have on the system, will vary according to the institutional characteristics of that system [16]. For instance, countries that attract many medical tourists could witness price increases and the diversion of services away from their less-fortunate citizens [1]. At the same time, the institutional features of national health care systems can explain why some countries attract more medical tourists than others. The comparison between Canada and the US is particularly revealing here. On the one hand, although some provinces have considered alternate approaches that would encourage inbound medical tourism as a source of revenue generation [52], at present the limited scope of private health care in Canada restricts the availability of medical tourism opportunities for wealthy foreigners seeking treatments. On the other hand, the large scope of private health care in the US makes that country an obvious target for wealthy medical tourists who can afford its high medical costs.

Second, and more important for this article, national health care institutions may also shape the way in which each country is affected by outbound medical tourism. For example, in a single-payer health care system such as Canada’s, both routine follow-up care and complications resulting from medical acts performed abroad are typically dealt with within the public system, engendering direct costs to taxpayers and potentially impacting access for others in the system (i.e., if physicians’ time is diverted to attend to emergent issues) [6]. The extent of these concerns varies depending on the urgency of the issue and whether it falls within hospital and physician services covered by the universal system (versus, for example, dental care where public coverage is more limited) [52]. By comparison, within the fragmented public-private US health care system, public programs may only absorb a fraction of the costs of complications related to outbound medical tourism, thus reducing their direct negative impact on taxpayers, whereas private insurance companies or individuals themselves might bear the majority of these costs.

The potential savings for outbound countries medical tourism generates are also likely to depend on the institutional features of each national or sub-national health care system [16]. In Canada, for instance, people who decide to go abroad for non-emergency surgeries might help reduce the length of waiting lists, although this positive impact might be limited by the fact that some of these surgeries are simply not available in Canada or, at least, not available to the individuals who seek treatments abroad (e.g., because of their age or health status). Because waiting lists are much less of an issue in the US [40], this potential benefit of medical tourism to domestic health care systems may be less relevant there.

Conversely, the prospect of affordable medical tourism may convince people in the US who do not have access to Medicaid, Medicare, or employer-based coverage that they do not need coverage at all, because they can always go abroad and save money should they need medical treatment. In this context, global medical tourism could interact with the question of whether people will seek coverage or not. At the same time, to save money, “US companies, such as Anthem Blue Cross and Blue Shield and United Group Programs, are now exploring the idea of including medical tourism as a part of their coverage,” a situation that could increase their administrative burden and create further complications along the road [53].

## Policy implications

Our aim with the preceding high-level overview was to draw on existing knowledge to highlight not only that national health care institutions may shape the demand for medical tourism in a particular country or region, but also that the consequences of such tourism for national health care systems are likely similarly mediated by the institutional features of these systems. These connections have a number of important potential implications for health system governance of medical tourism and, more specifically, for the options available to policy makers seeking particular objectives. For example, depending on the jurisdiction, efforts to reduce demand for medical tourism could include a range of options such as investing resources targeted at reducing domestic wait times, expanding public health insurance, limiting public coverage for follow-up care needs, or educating the public about the potential risks associated with medical tourism [2], among other options. Conversely, efforts to encourage the development of a medical tourism industry within a particular jurisdiction might involve regulatory change to expand options for private system offerings and targeted marketing campaigns, again among other possibilities [5, 17].

In fact, it has long been recognized the governments have a variety of tools or policy levers at their disposal when they seek to influence behavior [54]. Identifying which tool (or combination of tools) is likely to be most effective in a particular set of circumstances, such as medical tourism, requires a nuanced understanding of relevant institutional characteristics and situational factors. Accordingly, we propose that a comparative research agenda should be a key element of future analysis and decision-making efforts in this field. Such an agenda would not only help empirically test the above hypotheses about the institutional-medical tourism nexus, it could also help facilitate lesson drawing between jurisdictions that have attempted different approaches by helping pinpoint salient commonalities and points of difference between the systems that might initially explain, and ideally ultimately even predict, the likely results of particular policy initiatives.

## Research agenda

We propose a comparative research agenda that aims to explore the relationship between medical tourism and key institutional features of national health care systems. Although some aspects of our research agenda are already present in the existing literature, we think studying these elements together and with a comparative policy lens would be of tremendous value to health system decision -makers seeking to navigate different objectives including, for example, avoiding “brain drain” from public to private health care, minimizing added costs to publicly funded systems, protecting vulnerable individuals, and facilitating patient autonomy.

Drawing on our review of the health care systems in Canada and the US, we have identified three key institutional features that we suggest are particularly relevant to medical tourism and its broader policy context. These key features are health care funding models, delivery structures (e.g., public/private mix, provider payment models, role of user choice, and competition between providers), and governance systems (e.g., location of authority, health care provider regulation, liability systems). Future empirical research may identify other more salient features and certainly an iterative approach may be valuable. Nonetheless, we suggest that these features would provide a useful starting point for the next step, which we propose be an exploration of how these institutional features relate to the following areas:

(i) *Patient flow patterns* – e.g., inbound versus outbound, treatment destinations, types of treatment sought.
(ii) *Patient motivations* – e.g., cost reduction, wait list avoidance, pursuit of quality, circumvention tourism.
(iii) *Health system interactions* – e.g., costs and options for follow-up treatment, roles of domestic health care professionals.
(iv) *Existing policy levers* – e.g., public and private insurance structures, incentive schemes, information campaigns, regulation.

These four areas are not intended to serve as a comprehensive list of all relevant lines of enquiry. However, they present a valuable starting point, particularly because of their relevance to policy instrument selection processes. Having said that, and although it is beyond the scope of this piece to go further than laying a foundation for this proposed research agenda, we suggest that future research take a broad and scoping approach to draw on existing data and information and, where possible, conduct new empirical work addressing these critical areas. With a view to identifying patterns and generating hypotheses, researchers will likely need to continually refine the initial assumptions, outlined above, about the relationships between different institutional features and aspects of medical tourism. Doing so will require careful thought regarding the selection of an appropriate scientific paradigm, with a view to research validity and reliability [55].

We also anticipate that end-users and important stakeholders, including elected officials, civil servants, health care providers, and patients and families, would have an important contribution to make to the research design and with respect to interpreting the findings, particularly as they relate to the identification and evaluation of policy options. One important limitation in this type of work will relate to data availability. We expect that comparative work of this nature and any future empirical analyses it includes will highlight gaps in knowledge and potentially trigger future research agendas. Overall, the research envisioned here should complement and augment ongoing efforts in the field to improve understandings of important factors including patient flows, expenditure trends, system impacts, and individual decision-making determinants, among others.

## Conclusions

This article discussed the relationship between medical tourism and key institutional aspects of national health care systems with a view to highlighting the value in a comparative research agenda focused on identifying and evaluating policy options. First, we argued that these characteristics directly affect the demand for medical tourism in each country. Second, we suggested that such institutional characteristics shape the actual impact of medical tourism on that particular country**.** This discussion led to the formulation of an institutionalist research agenda about medical tourism. It is our hope that this proposed agenda will trigger discussion and debate, help develop future research, and inform new ways of thinking about medical tourism in the global landscape. Medical tourism is a complex phenomenon and we suggest that applying a comparative, institutional lens will shed new light on its drivers, constraints, and impacts and, in so doing, ultimately help inform policy development in this area.
